# The effect of intravenous lidocaine on propofol dosage in painless bronchoscopy of patients with COPD

**DOI:** 10.3389/fsurg.2022.872916

**Published:** 2022-09-15

**Authors:** Li Yang, Tao He, Min-Xiao Liu, Shi-Qiang Han, Zhi-Ang Wu, Wei Hao, Zhi-Xia Lu

**Affiliations:** ^1^ Department of Anesthesiology, Hebei Provincial Hospital of Traditional Chinese Medicine, Shijiazhuang, China; ^2^ Department of Radiotherapy, Hebei Provincial Hospital of Traditional Chinese Medicine, Shijiazhuang, China; ^3^ Department of Anesthesiology, Luquan Second People's Hospital, Shijiazhuang, China

**Keywords:** lidocaine, bronchoscopy, hypoxemia, propofol, sedation

## Abstract

**Background:**

We tested the hypothesis that intravenous (IV) lidocaine reduces propofol requirements in painless bronchoscopy in patients with chronic obstructive pulmonary disease (COPD).

**Methods:**

A total of 93 patients who underwent bronchoscopy were included in this randomized placebo-controlled study. The patients were randomly divided into two groups. After the IV doses of nalbuphine, patients were given a bolus of propofol, which was titrated if necessary until loss of consciousness. Then patients were given IV lidocaine (2 mg/kg then 4 mg/kg/h) or the same volume of saline. The primary endpoint was the propofol requirements. Secondary endpoints were the incidence of hypoxemia, the incidence of cough during glottis examination, the systolic blood pressure (SBP) and heart rate (HR) during bronchoscopy procedures, the bronchoscopist's comforts, and the time for wakefulness before recovery.

**Results:**

Lidocaine infusion resulted in a significant reduction in propofol requirements (*p* < .0001), and the incidence of hypoxemia (*p* = .001) and cough (*p* = .003) during examination decreased significantly in the lidocaine group. During the examination, the fluctuation of SBP and HR was significantly lower than that in the control group, and the difference was statistically significant (*p* < .05). Bronchoscopist's comforts were higher in the lidocaine group (*p* < .001), and time for wakefulness (*p* < .001) were significantly lower in the lidocaine group.

**Conclusion:**

In painless bronchoscopy in patients with COPD, IV infusion of lidocaine resulted in a reduction in propofol dose requirements and reduce the incidence of adverse events.

## Background

Chronic obstructive pulmonary disease (COPD) is a common and frequently occurring disease in Chinese elderly patients and most of whom require bronchoscopy. Bronchoscopy diagnosis and treatment is an operation with high stimulation intensity that causes strong discomfort for the patient. According to the current guidelines, painless bronchoscopy [sedation or intravenous (IV) anesthesia] is usually selected to improve the comfort and tolerance of patients (1). In painless bronchoscopy, midazolam and propofol are often combined with opioids which have the effect of central antitussive and can significantly inhibit the stimulation of airway operation. However, when propofol or midazolam is used alone for sedation and analgesia, the risk of respiratory and hemodynamic complications is as high as 10%–14.5% (2), and combined use will further increase the risk of hypoxemia and apnea (3). In order to reduce the dosage of sedative drugs, local anesthetics such as lidocaine are often injected into the upper respiratory tract and tracheobronchial tree through the working channel of a bronchoscope. If the airway is blocked by thick sputum, it may be difficult to make lidocaine evenly distributed by this method, resulting in incomplete anesthesia on the airway wall surface. IV lidocaine can not only reduce the cough response and pressor response during tracheal intubation (4) but also effectively reduce cough and hemodynamic changes during tracheal extubation (5). The mechanism by which lidocaine suppresses the cough reflex and causes hemodynamic changes is incompletely understood. But studies showed lidocaine may inhibit the cough reflex by inhibiting the brainstem, acting on peripheral upper airway receptors, or both (6). Therefore, in the painless bronchoscopy of patients with COPD, we combined IV lidocaine with propofol to verify the hypothesis that IV lidocaine reduces the dosage of propofol.

## Methods

### Participants

**Figure 1:**
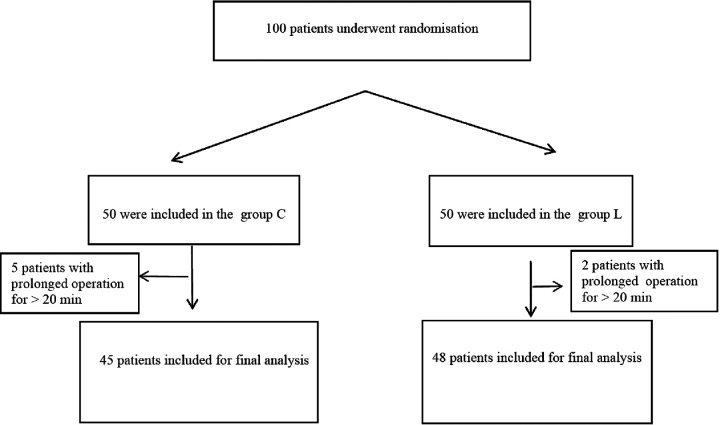
Consort flowchart.

According to the global initiative for chronic obstructive lung diseases (GOLDs), patients with a previous history of dyspnea, chronic cough, or sputum, post-bronchodilator forced expiratory volume in the first second/forced vital capacity (FEV_1_/FVC) < 0.7 and airflow restriction after inhalation of bronchodilators, and indications for bronchoscopy were selected as the research object. A total of 93 patients with COPD who underwent painless bronchoscopy from April 2019 to December 2019 in our hospital were included. SPSS software was used to generate a random assignment sequence, and 93 patients were randomly divided into two groups: the lidocaine group (group L, *n* = 48) and the control group (group C, *n* = 45). The randomly assigned sequences are placed in sequentially coded opaque sealed envelopes. After confirming that the patients met the inclusion criteria, the envelopes were distributed and opened according to the chronological order of patients' inclusion, then the patients were assigned to the corresponding groups and recorded. All the patients after screening were enrolled in the study (Figure 1).

After the patient entered the operating room, the anesthesiologist opened the sealed envelope and she was responsible for preparing the study drug. Another anesthesiologist was responsible for the intraoperative medication of all patients and did not know the grouping. Bronchoscopy doctors and nursing staff responsible for data collection did not know the grouping of patients as well.

### Criteria

(1) Inclusion criteria: 18–80 years old; ASA I–III; preoperative pulmonary function examination, FEV_1_/FVC < 70%; arterial blood gas PaO_2_ > 60 mmHg (air inhalation); consent to participate in the experiment and sign the informed consent form.
This study was approved by the ethics committee of Hebei Hospital of Traditional Chinese Medicine (2019-KY-004-02), and the registration number in the clinical trial registry was ChiCTR1900022178.
(2) Exclusion criteria: patients who did not agree to participate in painless treatment; COPD exacerbation or recent acute upper respiratory tract infection; severe cardiopulmonary disease before operation; allergic history of propofol and lidocaine hydrochloride; impaired verbal communication or mental disorder; examination time more than 20 min.

### Study design

**Figure 2:**
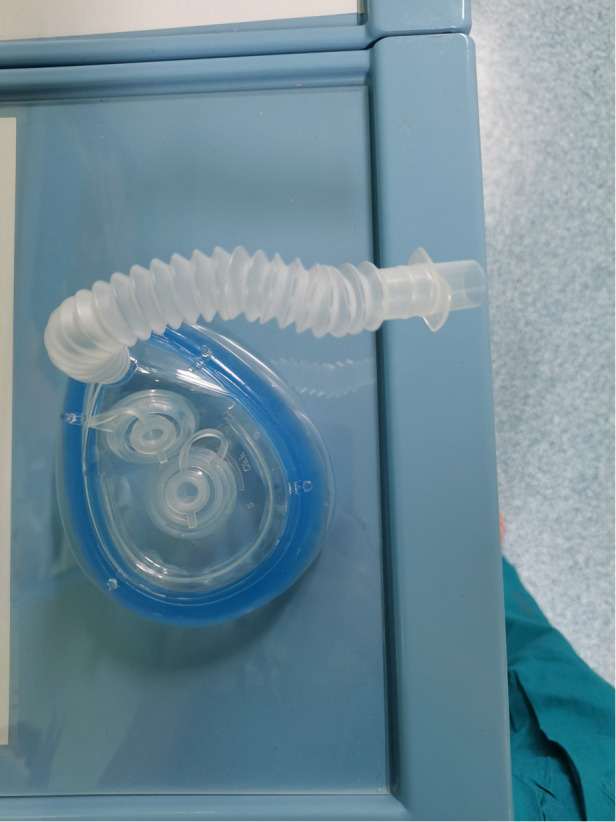
A special anesthesia mask.

Venous access was opened after entering the operating room and the patients were placed in a supine position. ECG, BP, and SpO_2_ monitoring were performed with a Mindrary9100 monitor (Shenzhen Mindray Bio-medical Electronics Co. LTD). Bronchoscopy procedures were performed transnasally, with the patients in the semi-recumbent position by three pulmonary fellow physicians under the close supervision of two pulmonary attending physicians. A special anesthesia mask (Henan Tuoren Medical Instrument Group Co. LTD, Figure 2) was used for oxygen inhalation, 6 l/min, and 1 ml of 2% lidocaine (H20059049, Ji Chuang Pharmaceutical Co., Taixing, China) was used for nasal inhalation.

### Intervention

Both groups of patients were injected with nalbuphine (H20130066, Ren Fu Pharmaceutical Co., Yichang, China) at 0.1 mg/kg intravenously, followed by propofol (H19990282, Li Bang Pharmaceutical Co., Xian, China) at 0.5 mg/kg intravenously. The patients were supplemented with propofol 10 mg/5 s to produce unconsciousness (the eyelash reflex disappeared and they did not respond) if necessary. Lidocaine group (group L): intravenously injected with a single dose of lidocaine at 2 mg/kg, followed by continuous pump injection at a speed of 4 mg/kg/h for 30 min, and toxic concentrations of lidocaine were not reached (2). Control group (group C): IV infusion of the same dose of normal saline. Before the bronchoscope entered the glottis, 2 ml of 2% lidocaine was sprayed on both sides of the piriform recess and glottis. If the patient has a severe cough, body moving and other conditions affecting the operation or hemodynamic changes [heart rate (HR) > 20 times/min, mean arterial pressure (MAP) > 20% of the basic value], stop the operation and inject propofol in 10 mg/5 s intravenously until the operation is carried out smoothly. During the examination, all patients breathed autonomously and inhaled oxygen (6 l/min) through the endoscope mask. When the blood pressure is lower than 20% of the basic value, phenylephrine is administered; when the HR is less than 45 beats/min, atropine is given; and when the arterial oxygen saturation is lower than 90%, jaw-lift and oxygen is supplied by pressure mask.

### Data collection

The dosage of propofol, the operation time, and the recovery time of patients (letting the patients open their eyes and if the patients can say their names correctly) were recorded after the operation. Records of whether severe cough (cough, labored breathing, and body moving are obvious to interfere with the operation) occurred in the glottis and during the whole operation. According to whether cough and body moving of patients affect the operation, bronchoscopists record the visual analog scale (VAS). A score of 0 means that the operation cannot be performed, and a score of 10 means that the operation is not affected at all.

### Outcomes

(1) Main outcome measures: induction dosage and total dosage of propofol.
(2) Secondary outcome measures: incidence of hypoxemia, incidence of cough in glottis and cough during the whole operation, systolic blood pressure (SBP) and HR at T0, T1, T2, T3, T4, VAS of bronchoscopy physician, recovery time.

### Statistical analysis

In the preliminary experiment, the dosage of propofol in the control group was 150 mg, with a standard deviation of 80, which is expected to reduce by 50 mg. The standard deviation was consistent with the control group, *α* = 0.05, power = 0.9. The sample size was calculated by PASS software, *n* = 45. Assuming a loss rate of 10%, a total of 100 patients were required.

SPSS 23.0 and GraphPad statistical software (GraphPad Prism version 5.0a, La Jolla, CA, USA) were used for data analysis and graphing. The normal distribution of the continuous variables was evaluated by the Shapiro–Wilk test. Measurement data of normal distribution were statistically described by means ± SD. Paired *t*-test was used for intra-group comparison, two independent sample *t*-test was used for inter-group comparison, and repeated measurement analysis of variance was used for blood pressure and HR. Categorical variables in the difference between groups were compared by using the *Chi*-square test. A *p*-value of <.05 indicated statistical significance.

## Results

In this study, a total of 93 cases were enrolled, of which 7 cases were excluded for operation times more than 20 min (2 cases in the lidocaine group and 5 cases in the control group).

### General data

There was no significant difference in age, weight, gender, lung function, ASA classification, smoking status, hypertension, and heart diseases between the two groups (*p* > .05, Table 1).

**Table 1:** Patient general information data.

General data	Group L (*n* = 48)	Group C (*n* = 45)	*p*
Age (years)	52.44 ± 12.82	51.40 ± 9.78	0.67
Weight (kg)	65.53 ± 6.8	65.16 ± 6.68	0.83
Male [*n* (%)]	16 (53.33)	18 (60.00)	0.60
ASA			
II	22 (73.33)	24 (80.00)	0.54
III	8 (26.67)	6 (20.00)	
Smoking status			
Yes	21 (70.00)	19 (63.33)	0.58
No	9 (30.00)	11 (36.67)	
Hypertension	18	20	0.73
Heart diseases	21	15	0.52
FEV_1_/FVC	51.63 ± 13.6	56.10 ± 11.3	0.17

FEV, forced expiratory volume; FVC, forced vital capacity.

### Propofol dosage, VAS, and recovery time

There was no significant difference in the average operation time between the two groups (*p* > .05). As shown in Table 2, the total amount of propofol in group L (103.96 mg ± 15.40 mg) was significantly lower than in group C (150.67 mg ± 50.25 mg) (*p* < .001). The difference is 46.70 mg (95% confidence interval: 62.388–31.028 mg). The recovery time of group L was significantly faster than that of the control group (*p* < .001), and VAS was higher than that of the control group (*p* < .001). These results suggest that using lidocaine before operation can reduce the amount of propofol used in operation, and can make patients recover better after operation.

**Table 2:** Comparison of propofol dosage, VAS, and recovery time between the two groups ($\bar{x}\pm s$).

	Propofol dosage (mg)	VAS	Recovery time (min)
Group L (*n* = 48)	104 ± 15	9.6 ± 0.17	4.13 ± 0.98
Group C (*n* = 45)	151 ± 50	8.3 ± 0.18	6.60 ± 1.86
95% confidence intervals for mean	(−61.81, −31.6)	(1.23, 1.37)	(−3.08, −1.87)
*t*-value	−6.14	5.28	−8.09
*p*-value	<.0001	<.0001	<.001

VAS, visual analog scale.

### Vital signs monitoring

**Figure 3:**
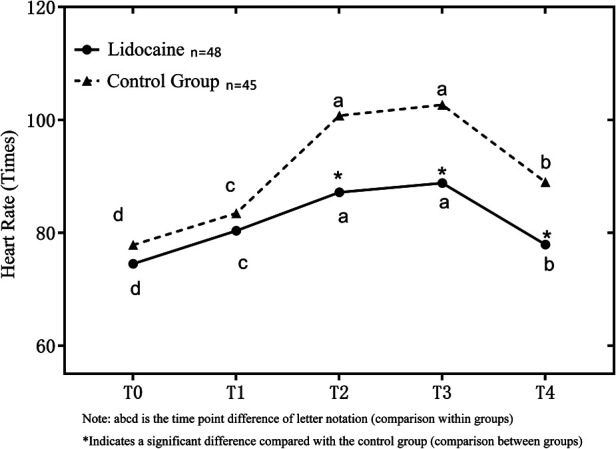
Comparison of heart rate (HR) between the two groups at different time points.

(1) SBP: There was no significant difference between the two groups at T0, T1, T2, and T3, but there was a significant difference in SBP at T4 (*p* < .05, Table 3).
(2) HR: There was no significant difference between the two groups at T0 and T1, but there was a significant difference at T2, T3, and T4 (*p* < .05, Figure 3). These may further confirm the advantages of IV lidocaine before bronchoscopy.

**Table 3:** Systolic blood pressure (SBP) of two groups at different time points ($\bar{x}\pm s$) compared with group C; ^a^*p* < .05.

SBP (mmHg)	T0	T1	T2	T3	T4
Group L (*n* = 48)	136.10 ± 17.96	144.54 ± 27.20	135.71 ± 30.29	143.25 ± 26.80	144.92 ± 16.17^a^
Group C (*n* = 45)	135.16 ± 17.10	142.60 ± 26.41	137.36 ± 19.62	133.53 ± 23.98	110.91 ± 12.05^a^

### Adverse events

The incidence of hypoxemia and cough in group L was lower than that in group C, and the difference was statistically significant (*p* < .05, Table 4), which means IV lidocaine can reduce the dosage of propofol and thus reduce the incidence of adverse reactions.

**Table 4:** Comparison of adverse event incidence between the two groups [case (%)].

Groups	SpO_2 _< 90%	Cough in glottis	Cough in operation
Group L (*n* = 48)	3 (6.3)	3 (6.3)	1 (2.1)
Group C (*n* = 45)	14 (31.1)	6 (13.3)	10 (22.2)
*χ* ^2^	10.67	1.33	9.03
*p*-value	.001	0.25	.003

## Discussion

This study investigated the effect of IV lidocaine on propofol dose requirements in patients with COPD during painless bronchoscopy. The results showed that the lidocaine group required less propofol than the control group. Our study demonstrates that IV infusion lidocaine could significantly result in a reduction in propofol needs for painless bronchoscopy without affecting the working conditions of the bronchoscope operator, and reducing hemodynamic fluctuations.

IV lidocaine has analgesic and antitussive effects (7–9), which is usually used to prevent cough during tracheal intubation and extubation (5, 10), and to reduce cardiovascular response during laryngoscopy and tracheal intubation (11, 12), and to significantly reduce the airway resistance in asthmatic patients (13). However, studies have shown that lidocaine reduces the incidence of cough during tracheal intubation, which is not because of deepening the depth of anesthesia (9). The mechanism of IV lidocaine on cough is still unclear, which may be related to the fact that lidocaine can inhibit the excitement of airway C-fiber receptors (14), selectively inhibit pain conduction at the spinal cord level (15), and reduce tonic discharge of active peripheral nerve fibers (9, 16). Continuous IV infusion of lidocaine can reduce the dosage of propofol during total IV anesthesia (17), which can only be observed during surgical stimulation, indicating that lidocaine has the characteristics of anti-injury response (18). Recently, it has been shown that patient-controlled sedation may be advantageous in bronchoscopy, especially using propofol combined with lidocaine (19–20), and our study further confirmed this conclusion in a population of COPD patients requiring bronchoscopy. Therefore, we applied lidocaine to painless bronchoscopy, which not only greatly reduced the dosage of propofol but also reduced the incidence of cough. However, there was no difference in the incidence of coughing in the glottic between the two groups, which may be related to sufficient superficial anesthesia of bilateral piriform recess and glottic with 2% lidocaine.

In this study, the fluctuation of SBP and HR in group L was significantly smaller than that in group C, but the fluctuation of SBP was smaller than that of HR, which may be due to the fact that bronchoscopy in the supine position stimulates the sympathetic nervous system and has a greater impact on HR (21). At the end of the examination, the blood pressure of group C was significantly lower due to the larger dosage of propofol, and the patient's recovery was significantly slower than that of group L.

It should be noted that the reduction in propofol dosage was not at the expense of working conditions, because VAS in group L was higher than that in group C.

In our study, the incidence of hypoxemia in group L was significantly reduced. In painless bronchoscopy, respiratory depression is a common complication (22). The purpose of IV lidocaine is to reduce the dosage of propofol and thus reduce the incidence of adverse reactions. Therefore, continuous IV infusion of lidocaine combined with propofol has less respiratory depression and fewer adverse respiratory events.

There are some limitations to this study. First, hypoxemia was monitored by SpO_2_. However, SpO_2_ may be a late indicator of hypoventilation. In previous studies, we found that the median delay from apnea to significant SpO_2_ decline was 31 s (21). By monitoring end-tidal carbon dioxide (ETCO_2_) during bronchoscopy, we can detect under ventilation earlier. Second, when retaining spontaneous breathing, severe airway stimulation can not be completely inhibited, and laryngeal mask anesthesia is required for tumor resection or rigid bronchoscopy. Besides, pre-experiment results showed that most patients with COPD took less than 20 min of painless bronchoscopy, so we excluded those patients with more than 20 min. Therefore, this study can only be applied to the diagnosis and treatment of short bronchoscopy (more than 20 min were excluded), such as bronchoalveolar lavage, brushing, and sputum suction.

## Conclusion

In conclusion, in the painless bronchoscopy of COPD patients, continuous IV infusion of lidocaine can significantly reduce the dosage of propofol, and reduce the incidence of cough and hypoxemia.

## Data Availability

The original contributions presented in the study are included in the article/Supplementary Material, further inquiries can be directed to the corresponding author/s.

## References

[1] Du Rand IA Blaikley J Booton R Chaudhuri N Gupta V Khalid S British Thoracic Society Guideline for diagnostic flexible bronchoscopy in adults: accredited by NICE. Thorax. (2013) 68:i1–44. 10.1136/thoraxjnl-2013-203618 23860341

[2] McQuaid KR Laine L. A systematic review and meta-analysis of randomized, controlled trials of moderate sedation for routine endoscopic procedures. Gastrointest Endosc. (2008) 67:910–23. 10.1016/j.gie.2007.12.046 18440381

[3] Qadeer MA Vargo JJ Khandwala F Lopez R Zuccaro G. Propofol versus traditional sedative agents for gastrointestinal endoscopy: a meta-analysis. Clin Gastroenterol Hepatol. (2005) 3:1049–56. 10.1016/S1542-3565(05)00742-1 16271333

[4] Thompson Kate R Eva R. Effects of intravenous and topical laryngeal lidocaine on heart rate, mean arterial pressure and cough response to endotracheal intubation in dogs. Vet Anaesth Analg. (2016) 43:371–8. 10.1111/vaa.12303 26484728

[5] Hu S Li Y Wang S Xu S Ju X Ma L. Effects of intravenous infusion of lidocaine and dexmedetomidine on inhibiting cough during the tracheal extubation period after thyroid surgery. BMC Anesthesiol. (2019) 19:66–74. 10.1186/s12871-019-0739-1 31054568 PMC6500031

[6] Stolz D Kurer G Meyer A Chhajed PN Pflimlin E Strobel W Propofol versus combined sedation in flexible bronchoscopy: a randomised non-inferiority trial. Eur Respir J. (2009) 34(5):1024–30. 10.1183/09031936.00180808 19386684

[7] Dunn LK Durieux ME. Perioperative use of intravenous lidocaine. Anesthesiology. (2017) 126:729–37. 10.1097/ALN.0000000000001527 28114177

[8] Panti A Cafrita IC Clark L. Effect of intravenous lidocaine on cough response to endotracheal intubation in propofol-anaesthetized dogs. Vet Anaesth Andanalgesia. (2016) 43:405–11. 10.1111/vaa.12332 26671878

[9] Clivio S Putzu A Tramèr MR. Intravenous lidocaine for the prevention of cough: systematic review and meta-analysis of randomized controlled trials. Anesth Analg. (2019) 129:1249–55. 10.1213/ANE.0000000000003699 30169416

[10] Qi DY Wang K Zhang H Du BX Xu FY Wang L The efficacy of intravenous lidocaine versus placebo on attenuating cardiovascular response to laryngoscopy and tracheal intubation: a systematic review of randomized controlled trials. Minerva Anestesiol. (2013) 79:1423–35. 10.1213/ANE.0b013e3182a9ac39 23839320

[11] Vivancos GG Klamt JG Garcia LV. Effects of 2 mg.kg^−1^ of intravenous lidocaine on the latency of two different doses of rocuronium and on the hemodynamic response to orotracheal intubation. Braz J Anesthesiol. (2011) 61:1–12. 10.1016/S0034-7094(11)70001-0 21334502

[12] Adamzik M Groeben H Farahani R Lehmann N Peters J. Intravenous lidocaine after tracheal intubation mitigates bronchoconstriction in patients with asthma. Anesth Analg. (2007) 104:168–72. 10.1213/01.ane.0000247884.94119.d5 17179265

[13] Burki NK Lee LY. Blockade of airway sensory nerves and dyspnea in humans. Pulm Pharmacol Ther. (2010) 23:279–82. 10.1016/j.pupt.2010.02.002 20188847 PMC2885590

[14] Tanelian DL MacIver MB. Analgesic concentrations of lidocaine suppress tonic A-delta and C fiber discharges produced by acute injury. Anesthesiology. (1991) 74:934–6. 10.1097/00000542-199105000-00020 2021210

[15] Woolf CJ Wiesenfeld-Hallin Z. The systemic administration of local anaesthetics produces a selective depression of C-afferent fibre evoked activity in the spinal cord. Pain. (1985) 23:361–74. 10.1016/0304-3959(85)90006-5 3937116

[16] Guo YH Zhao CF. Effect of lidocaine on half effective dose of propofol induced unconsciousness. J Clin Anesthesiol. (2016) 32(02):189–90. CNKI:SUN:LCMZ.0.2016-02-027

[17] Altermatt FR Bugedo DA Delfifino AE Solari S Guerra I Muñoz HR Evaluation of the effect of intravenous lidocaine on propofol requirements during total intravenous anaesthesia as measured by bispectral index. Br J Anaesth. (2012) 108:979–83. 10.1093/bja/aes097 22490315

[18] Ali AH Toba H Sakiyama S Yamamoto R Takizawa H Kenzaki K Holter ECG monitoring of sympathovagal fluctuation during bronchoscopy. Clin Respir J. (2016) 10(2):204–10. 10.1111/crj.12204 25195956

[19] Forster C Vanhaudenhuyse A Gast P Louis E Hick G Brichant JF Intravenous infusion of lidocaine significantly reduces propofol dose for colonoscopy: a randomised placebo-controlled study. Br J Anaesth. (2018) 121(5):1059–64. 10.1016/j.bja.2018.06.019 30336850

[20] Kelsaka E Karakaya D Baris S Sarihasan B Dilek A. Effect of intramuscular and intravenous lidocaine on propofol induction dose. Med Princ Pract. (2011) 20(1):71–4. 10.1159/000319761 21160218

[21] Wood-Baker R Burdon J McGregor A Robinson P Seal P. Fibre-optic bronchoscopy in adults: a position paper of The Thoracic Society of Australia and New Zealand. Intern Med J. (2001) 31(8):479–87. 10.1046/j.1445-5994.2001.00104.x 11720062

[22] Ishiwata T Tsushima K Fujie M Suzuki K Hirota K Abe M End-tidal capnographic monitoring to detect apnea episodes during flexible bronchoscopy under sedation. BMC Pulm Med. (2017) 17:7. 10.1186/s12890-016-0361-7 28061836 PMC5219680

